# Correction of misaligned slices in multi-slice cardiovascular magnetic resonance using slice-to-volume registration

**DOI:** 10.1186/1532-429X-10-13

**Published:** 2008-02-29

**Authors:** Adam G Chandler, Richard J Pinder, Thomas Netsch, Julia A Schnabel, David J Hawkes, Derek LG Hill, Reza Razavi

**Affiliations:** 1 King's College London, Division of Imaging Sciences, St Thomas' Hospital, London, UK; 2 Philips Research, Hamburg, Germany; 3 Centre for Medical Image Computing, University College London, UK

## Abstract

A popular technique to reduce respiratory motion for cardiovascular magnetic resonance is to perform a multi-slice acquisition in which a patient holds their breath multiple times during the scan. The feasibility of rigid slice-to-volume registration to correct for misalignments of slice stacks in such images due to differing breath-hold positions is explored. Experimental results indicate that slice-to-volume registration can compensate for the typical misalignments expected. Correction of slice misalignment results in anatomically more correct images, as well as improved left ventricular volume measurements. The interstudy reproducibility has also been improved reducing the number of samples needed for cardiac MR studies.

## Purpose

Medical imaging has an important role in the management of patients with heart disease. Cardiovascular magnetic resonance (CMR) is currently the gold standard for the assessment of ventricular function [1]. One of the main challenges with cardiac image acquisition is to account for cardiac motion due to respiration, which can lead to severe artefacts in the final images. Numerous techniques for motion compensation have been proposed for use with CMR acquisitions [2], including respiratory gating, and model based methods; however poor reproducibility of the respiratory cycle and the complex motion of the heart with respiration [3] means that breath-holding is still the most reliable and widely used approach.

A popular technique to reduce respiratory motion is to perform a multi-slice acquisition in which a patient holds their breath multiple times during the scan. It is not possible to acquire sufficient slices to cover the entire heart in a single breath-hold, even by using the latest improvements in acquisition technology such as Steady State Free Precession (SSFP) imaging [4,5] and sensitivity encoding (SENSE) [6,7].

In view of the spatial and temporal resolution needed for the imaging of the heart, it is only possible to acquire between one and three slices in a single breath-hold. The most common and important sequence currently used in the assessment of ventricular function by CMR is the short axis (SA) SSFP cine stack image, which usually consists of 10–14 parallel slices and around 20 cardiac phases. During the acquisition of the SA stack image, the subject is asked to hold their breath multiple times at the same breath-hold position, with between one and three slices acquired per breath-hold. The major drawback with such a method is that the integrity of the image volume depends greatly on how consistently a subject can hold his or her breath at the same position. If the position of the heart, in the different breath-holds, is very different there will be errors introduced when the images are analysed. The accuracy and reproducibility of SA SSFP imaging is very important as this defines the number of subjects needed in research studies of cardiac function that use imaging end-points. This is where CMR has a considerable advantage over echocardiography and improving reproducibility will have a large impact on the size and cost of studies of ventricular function in, for example, looking at drug therapy of heart failure. Although fast under-sampling acquisition techniques [8] have been developed to try to obtain similar spatial and temporal resolution to SA SSFP images in a single short breath-hold, they have not yet been shown to provide equivalent information, and are not in clinical use.

Recently it has been possible to acquire three-dimensional (3D) SSFP volumes of the heart covering 1–3 cardiac phases in a single breath-hold. This is done in a straight axial plane and produces 3D volumes of the heart with good spatial resolution. These volumes provide high quality anatomical information, but insufficient temporal resolution for the analysis of cardiac function. Using these 3D volumes, we believe that retrospective slice-to-volume image alignment is capable of correcting for the misalignments caused by differing breath-hold positions in the SA image.

Only a few techniques have been published suggesting how to align SA slice stacks with respect to each other [9-11]. The technique suggested by Lotjonen [10] and Moore [9] only corrected the translational movement of the heart caused by different breath-hold positions and the technique suggested by Swingen [11] only allowed for in-plane movement to be corrected. Such correction techniques may not be sufficient because previous work has shown that respiration causes both translations and rotations of the heart in all three dimensions [3]. The proposed slice-to-volume technique has the potential to correct for the expected 3D translational and rotational movement of the stacks relative to each other due to differing breath-hold positions.

## Materials and Methods

The aim of this paper was to correct for misaligned cardiac anatomy in multi-slice SA images by rigidly registering stacks of two slices to a high-resolution 3D MR axial cardiac volume. The first study was a simulation experiment, in which the accuracy and robustness of the registration was assessed. The second study corrected a truly misaligned multi-slice, multi-phase SA examination. Finally, an interstudy reproducibility study was performed to determine whether slice-to-volume registration could be used to obtain more reproducible measures of ventricular function from CMR SA slices. This could be beneficial in improving accuracy and reducing the number of subjects needed to confirm the statistical significance of a particular result.

Image registration is commonly defined as determining the geometrical transformation (or mapping) between two or more images such that after registration, corresponding features in the images are aligned. The transformation will have a number of degrees of freedom suitable to the application, e.g.: 6 degrees of freedom for a rigid body transformation in 3D, and thousands of degrees of freedom for some non-rigid registration methods. For this particular application a rigid transformation was used.

A rigid transformation for a 3D image is composed of 6 degrees of freedom: a translation in the x, y, and z directions and rotations *α*, *β*, *γ *about these three axes. A rigid-body transformation matrix *T*_*rigid *_can be represented as a rotation *R *followed by a translation *t *= (t_*x*_, t_*y*_, t_*z*_)^T ^which can transform a point *x *in the image:

*T*_*rigid *_(*x*) = *Rx *+ *t*

where the rotation matrix *R *is:

$$R=\left(\begin{array}{ccc} \cos\beta \cos\gamma & \cos\alpha \sin\gamma +\sin\alpha \sin\beta \cos\gamma & \sin\alpha \sin\gamma -\cos\alpha \sin\beta \cos\gamma \\ -\cos\beta \sin\gamma & \cos\alpha \cos\gamma -\sin\alpha \sin\beta \sin\gamma & \sin\alpha \cos\gamma +\cos\alpha \sin\beta \sin\gamma \\ \sin\beta & -\sin\alpha \cos\beta & \cos\alpha \cos\beta \end{array}\right)$$

The majority of medical image registrations are performed on 3D image volumes known as 3D-3D registration, although 2D-2D, 2D-3D (projection-to-volume and slice-to-volume) studies have also been performed [12,13]. Slice-to-volume registration has been applied to the brain for the applications of functional MR imaging [14], post-mortem pathology studies [15], and scout scan planning [16] and to abdominal organs, in particular the prostate [17] and liver [18] for interventional guidance. Slice-to-volume registration has also been performed on CMR data in which a porcine model was used to study the accuracy of affine alignment of different cardiac phases acquired at a single fixed breath-hold position [19]. In recent years, a number of fully automated intensity based registration techniques that use an information-theoretic measure, in particular Normalized Mutual Information (NMI) [20], has been shown to be amongst the most accurate for a variety of intra and intermodality medical image registration problems. NMI assumes a statistical relationship between the intensities in the images to be registered. Traditionally, a similarity measure that assumes an identity or linear relationship between the intensities in the images to be registered, such as Sum of Squared Differences (SSD) or Cross Correlation, would be used for intramodality registration problems. For the particular registration task in this paper, the degree of partial volume effect in the SA slices is severe and an identity/linear relationship between the intensities in the SA slice stacks and 3D volume cannot be assumed. For this reason, NMI is the similarity measure used in this paper to rigidly register the stacks of the SA images to the high-resolution 3D MR axial cardiac volume.

A simple iterative optimisation scheme, using 10 steps at 3 different image resolutions, was used to optimise the registration algorithm. The benefits of performing a multi resolution optimisation are that the speed of the registration algorithm is increased as well as an improvement in the robustness of the algorithm to local maxima and minima in the similarity space. The two images to be registered are re-sampled in all three dimensions by a factor of 2 and 4 to produce images with 1/2 and 1/4 of their original number of voxels respectively. The optimisation is initially performed at the coarsest resolution level. Each of the 6 transformation parameters is altered in turn by a particular step size and a value of the similarity measure calculated. The algorithm then moves the current estimate of the solution in the direction that produced the greatest improvement in the similarity measure. This is repeated until no improvement in the similarity measure is achieved. The initial step size chosen for the coarsest resolution level was 16 mm or 16 degrees. The step size is then reduced by a factor of 2 and the process repeated. This is repeated 10 times until a step size of 0.03125 mm or 0.03125 degrees is used. The complete optimisation scheme is then repeated for the images with twice the resolution of the original images with an initial step size of 8 mm and 8 degrees and finally at the original resolution level with an initial step size of 4 mm or 4 degrees.

### Simulation experiment

For this experiment, a misaligned SA image was simulated and slice-to-volume registration was used to try to recover the misalignments. Two data sets were acquired on a Philips Intera 1.5T I/T using a 5-element cardiac array coil. The first was a high-resolution end-diastolic 3D SSFP axial CMR volume (TE 1.4 ms, TR 3.2 ms, slice thickness 1.52 mm, number of slices 50, field of view 39 × 39 cm, read matrix 256, phase matrix 256, flip angle 45°, acquisition time 25s), and the second was a CMR multi-slice SA image (TE 1.6 ms, TR 3.3 ms, slice thickness 8 mm, number of slices 12, field of view 32 × 32 cm, read matrix 256, phase matrix 256, frames 25, temporal resolution 23 ms, flip angle 50°, 2 of the 12 slices acquired per breath-hold, average acquisition time per breath-hold 15s, total acquisition time 120s).

In this simulation, the multi-slice SA image was acquired from a very cooperative and experienced volunteer who could reproducibly hold his breath at the same position for all six breath-holds in the study. This image was treated as an approximately motion-free image, so that the positions of the 6 slice stacks (containing two slices per stack) could be used as gold standard positions for the subsequent simulation experiment. A cardiologist visually inspected the motion-free SA acquisition and indicated that the 6 different stacks were well aligned. To quantify this, each stack from the end diastolic phase was rigidly registered to the 3D volume. It was found that the original positions of the 6 slice stacks were within sub-voxel accuracy (0.80 ± 0.26 mm) of the correctly aligned position. This was accurate enough to be able to use them as a gold standard to validate the results from the simulation experiment.

Segmentation was used to remove regions within the SA image that were not of interest, such as the chest wall and spine. We then artificially misregistered the 6 stacks from the SA end-diastolic phase image by random amounts to allow us to simulate a subject holding their breath at relatively different positions for each breath-hold. For simulation of realistic breath-hold positions, the results from a study of the motion of the heart due to respiration [3] were used to determine the range of the rigid misregistration transformation applied. Although McLeish [3] found there to be rigid and non-rigid deformations of the heart due to respiration, we will only consider the correction of rigid degrees of freedom in the paper. This issue will be discussed further in the conclusion. The ranges of the translation in the x, y and z directions were ± 1.9 mm, ± 3.6 mm, and ± 12.2 mm respectively, and the ranges of the rotations in the x, y and z directions were ± 0.8 degrees, ± 3.2 degrees, and ± 0.4 degrees respectively. For each of the 6 stacks, 20 random misregistrations constrained by these ranges were applied. The end-diastolic phase of the simulated misaligned slice stacks were then rigidly registered (using the NMI similarity measure) to the end-diastolic phase image of the high-resolution 3D MR axial cardiac volume to try to recover the applied misregistrations.

By using the end-diastolic phase (the phase of the heart where least motion occurs) of the multi-slice SA image and the 3D volume image, the differences in cardiac anatomy due to heart deformation over the cardiac cycle could be ignored. This allowed us to correct for just the change in position of the heart due to differing breathhold positions using a rigid body transformation.

### Correction of truly misaligned SA images

Slice-to-volume registration was used to try to recover misalignment of SA slice stacks acquired from a volunteer who had moved between breath-holds (we refer to this as "truly misaligned"). For this experiment, the two images described in the previous subsection were used in addition to a truly misaligned SA image (TE 1.6 ms, TR 3.3 ms, slice thickness 8 mm, number of slices 12, field of view 32 × 32 cm, read matrix 256, phase matrix 256, frames 25, temporal resolution 23 ms, flip angle 50°, 2 of the 12 slices acquired per breath-hold, average acquisition time per breath-hold 15s, total acquisition time 120s) in which the individual acquisitions are misaligned by asking the volunteer to hold their breath at relatively different exhale positions for each of the 6 breath-holds.

The 6 stacks from the end-diastolic phase of the misaligned SA image were segmented to exclude non-cardiac tissue and rigidly registered separately to the end-diastolic phase of the 3D SSFP volume in order to correct the misalignment of the stacks for the end-diastolic cardiac phase. For each slice stack acquisition (15s duration), we made the assumption that the position of the breath-hold remained constant and subsequently all cardiac phases were equally misaligned. Therefore, to realign the other 24 phases of the SA image, the resultant rigid transformation obtained for each of the end-diastolic stacks was applied to the corresponding misaligned stack in the other 24 phases.

### Interstudy reproducibility study

This experiment investigated whether slice-to-volume registration could improve the interstudy reproducibility of left ventricular function studies from SA CMR. Ten healthy volunteers were used in this study. The subject group consisted of 4 women and 6 men. For each volunteer, two MR multi-slice SA images (TE 1.5 ms, TR 3.0 ms, slice thickness 8 mm, number of slices 12–14, field of view 43 × 43 cm, read matrix 256, phase matrix 256, frames 20, temporal resolution 40 ms, flip angle 50°, 2 of the 12–14 slices acquired per breath-hold, average acquisition time per breath-hold 15s, total acquisition time 120s) were acquired as well as a high-resolution end-diastolic 3D SSFP axial cardiac MR volume (TE 1.4 ms, TR 3.2 ms, slice thickness 1.52 mm, number of slices 50, field of view 39 × 39 cm, read matrix 256, phase matrix 256, flip angle 45°, acquisition time 25s).

For each volunteer, the 6–7 stacks from the end-diastolic phase of each of the two SA images were roughly segmented and rigidly registered separately to the end-diastolic phase of the 3D SSFP volume in order to correct for the misalignment of the stacks. Once the rigid body transformations were obtained for each end-diastolic stack, they were used to realign the corresponding misaligned stack in the end-systolic and end-diastolic phases of the SA images. To produce the SA images after registration, each of the misaligned stacks were transformed using the registration transform into a regularly spaced voxel grid (256 × 256, 12 slices, 1.25 × 1.25 mm^2 ^in-plane sampling by 8 mm slice spacing) using linear interpolation to fill in areas where data was missing. This resulted in a realigned end-diastolic and end-systolic SA image for each of the two originally acquired SA images.

For each dataset, the end-diastolic left ventricular (LV) volume (V_ed_) and end-diastolic LV volume (V_es_), was determined by means of manual segmentation performed by an expert using the Analyze software (Mayo Clinic, Rochester). The ejection fraction (EF) of the LV was then calculated using the following formulation:

$$EF=\frac{V_{ed}-V_{es}}{V_{ed}}$$

This was performed on the two SA images before slice-to-volume registration as well as the two realigned SA images.

Table 1 shows the mean, standard deviation (SD), minimum and maximum values of the V_ed_, V_es _and EF over the volunteer group for the SA images before registration as well as the realigned SA images.

**Table 1:** The mean, SD, minimum and maximum values of the V_ed_, V_es _and the EF for the SA images before and after slice-to-volume registration.^1^

	**The mean ± SD (min-max) over the group**		
	**V_ed_(ml)**	**V_es_(ml)**	**EF (%)**
**SA images before registration**	153 ± 29 (108–178)	52 ± 12 (30–70)	66 ± 6 (54–74)
**SA images after registration**	152 ± 28 (108–186)	45 ± 7 (29–55)	70 ± 5 (60–75)

^1^SD = standard deviation, SA = short axis, V_ed _= end-diastolic left ventricular volume, V_es _= end-systolic left ventricular volume, EF = ejection fraction.

For each volunteer, the difference in the V_ed_, V_es _and EF was calculated between the two SA images before slice-to-volume registration as well as the two realigned SA images. These difference values were then averaged over the total volunteer group (the mean difference) and the SD of the mean difference calculated. The SD of the mean difference is effectively the interstudy reproducibility of the measure in question.

The sample sizes required to show a clinical difference (*δ*) with a power (*P*) of 90% and an *α *error of 0.05 were also calculated for the SA images before slice-to-volume registration and after [21]:

*n *= *f *(*α*, *P*)·*σ*^2^·2/*δ*^2^

where *n *is the sample size, *P *is the study power required (the probability of detecting a significant event), *α *is the significance level, and *f *the value of the factor for different values of *α *and *P *(*f *= 10.5 for *α *= 0.05 and *P *= 0.90). *σ *is the interstudy reproducibility of the measure in question (the SD of the mean difference over the study group) and *δ *is the desired difference to be detected. For this study, the desired difference to be detected for V_ed _and V_es _was a 10 ml change and for the EF was an absolute change of 3%. These differences are in accordance with previous studies [22-25] to allow for a direct comparison of results.

## Results

### Simulation experiment

To evaluate the accuracy and robustness of the simulated experiment, the *mean imaged patient voxel displacement *(MIPVD) [26] was used to determine how well the registration recovered the 20 misregistrations for each of the 6 stacks. The 6 stack positions of the motion-free MR multi-slice SA image were used as a gold standard. This means that a successful recovery of the misregistration would yield a small MIPVD. The mean MIPVD before registration for all 20 random starting estimates for all 6 stacks was 7.01 ± 0.02 mm, and after registration this was reduced to 1.56 ± 0.49 mm. These results suggest that slice-to-volume registration is sufficiently robust and accurate within sub-voxel accuracy to recover the expected misalignments of slice stack position in a realistic situation.

### Correction of truly misaligned SA images

In order to see how accurately slice-to-volume registration has corrected the misalignment of the 6 stacks in the misaligned SA image, alignment of the left ventricular wall was visually compared before and after slice-to-volume registration, to the left ventricular anatomy obtained for the motion-free SA image (see Fig. 1). It can be seen that the LV wall of the misaligned SA image before registration shows significant displacement due to misregistration. The wall of the LV of the misaligned SA image after registration depicts a smoother ventricular surface, very much like the left ventricular surface obtained from the motion-free image. To confirm how well the registration performed, slices of the motion-free SA image were compared, by means of difference images, to the corresponding slice positions of the misaligned SA image before and after registration for all 25 cardiac phases. The SA image after registration was constructed the same as described in the previous section. The results for 3 of the 25 phases are shown for a slice from the centre of the LV in Fig. 2.

**Figure 1:**
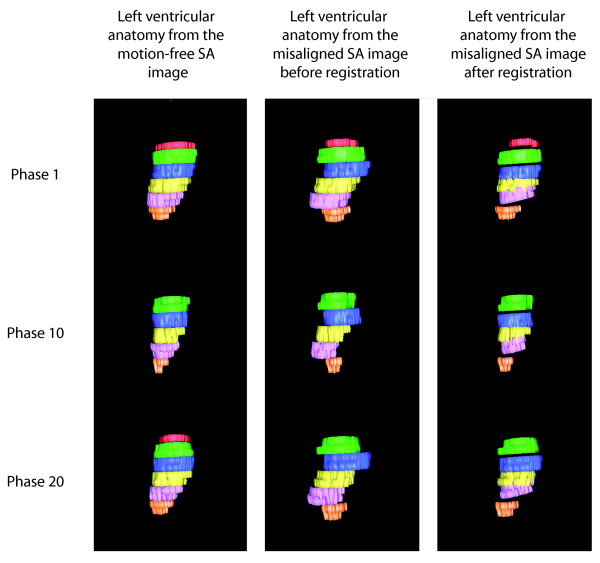
Visualization of the left ventricular wall from the motion-free SA image, and the misaligned SA image before and after registration respectively, for 3 of the cardiac phases. The different colours represent the 6 stacks acquired at different breath-holds.

**Figure 2:**
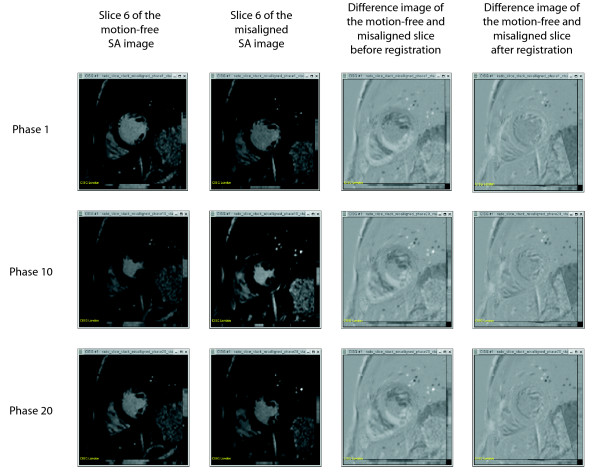
Columns 1 and 2 show a slice of the motion-free and misaligned SA image for 3 cardiac phases. Column 3 shows the difference image between the motion-free and misaligned SA slice before registration, and column 4 shows the difference image between the motion-free and misaligned slice after registration.

From Fig. 2, it can be seen that when the anatomy within the heart region before and after rigid slice-to-volume registration is compared to the motion-free SA image, the registration achieves an anatomically more correct image. To confirm this the SSD between the motion-free SA image and the misaligned SA image, before and after registration, was calculated for each of the 25 phases within the area of the heart. For all phases, the SSD of the motion-free SA image and the misaligned SA image after registration was always less (by 43.5% on average) than that of the motion-free SA image and the misaligned SA image before registration. This implies that the misaligned SA image after registration is more similar to the motion-free SA image than before registration.

As an alternative validation of the techniques, the accuracy of the LV volume over the cardiac cycle was calculated from the misaligned SA image before and after slice-to-volume registration. In both cases, the LV volume measurements (obtained in the same way as described in the previous section) were compared to measurements calculated from the motion-free SA acquisition on the same subject. On average, the misaligned volume deviates from the motion-free volume by 5.8 ml with a standard deviation of 4.1 ml whereas the realigned volume only deviates by 3.2 ml with a smaller standard deviation of 2.7 ml. This is further confirmation that the slice-to-volume registration has recovered the misalignment due to differing breath-hold positions.

### Interstudy reproducibility study

The mean difference ± SD calculated for the measures of V_ed_, V_es_, and the EF for repeated cardiac SA images before and after slice-to-volume registration can be seen in Table 2. The values of the mean difference ± SD before slice-to-volume registration are in accordance with previous studies [22-25].

**Table 2:** The mean difference ± SD of the V_ed_, V_es _and the EF for repeated scans of the SA images before and after slice-to-volume registration.^1^

	**The mean difference ± SD between repeated scans**		
	**V_ed_(ml)**	**V_es_(ml)**	**EF (%)**
**SA images before registration**	8.6 ± 5.8	3.0 ± 3.4	2.8 ± 1.9
**SA images after registration**	5.7 ± 4.2	3.4 ± 1.8	2.2 ± 1.3

^1^SD = standard deviation, SA = short axis, V_ed_ = end-diastolic left ventricular volume, V_es_ = end-systolic left ventricular volume, EF = ejection fraction.

By using slice-to-volume registration to correct for misalignments of SA stacks, the SD (the interstudy reproducibility) of the mean difference over the study group is reduced for all measures. Using equation 2, the sample sizes were calculated to show a clinically significant change in the V_ed _and V_es _of 10 ml, and an absolute change of 3% in the EF. These results can be seen in Table 3. The numbers in the brackets of Table 3 are the sample sizes rounded up to the nearest whole figure because fractions of samples are not possible. The reduction in sample size in Table 3 was calculated using the following equation:

**Table 3:** The SD and required sample size to show a clinical change with a power of 90% and an *α *error of 0.05 to detect a change of the V_ed _and V_es _of 10 ml and an absolute change in the EF of 3% for SA images before and after registration.^1^

	**SA images before registration**		**SA images after registration**		
	**SD**	**Sample size**	**SD**	**Sample size**	**Reduction in sample size**
**10 ml change in V_ed_**	5.8	7.1 (8)	4.2	3.7 (4)	48%
**10 ml change in V_es_**	3.4	2.4 (3)	1.8	0.7 (1)	71%
**3% absolute change in EF**	1.9	8.4 (9)	1.3	3.9 (4)	53%

^1^SD = standard deviation, SA = short axis, V_ed _= end-diastolic left ventricular volume, V_es _= end-systolic left ventricular volume, EF = ejection fraction.

$$\text{Reduction in sample size}=1-\frac{{\left(\text{SD of the mean difference after registration}\right)}^{2}}{{\left(\text{SD of the mean difference before registration}\right)}^{2}}\times 100$$

By successfully reducing the interstudy reproducibility with slice-to-volume registration, a reduction in study sample size of 48% for the V_ed_, 71% for the V_es _and 53% for EF could be achieved assuming that the interstudy reproducibility is the main source of error in the study.

## Conclusion

This work has shown that the slice-to-volume registration is sufficiently accurate and robust to realign misaligned SA cardiac stacks for improved visualizations and more accurate LV function analysis. Furthermore, it has been shown that the registration algorithm is robust enough to recover typical movements expected in misaligned slice stack positions. Such improvements will be beneficial for diagnosis and treatment planning for historic data as well as for data acquired when the patient is unable to hold their breath at a consistent position. It was also demonstrated that by using slice-to-volume registration to correct for misalignments in SA images, one could improve the interstudy reproducibility and hence reduce the number of samples needed for CMR studies to show the same statistical significance. The interstudy reproducibility is a pivotal factor for determining the ability of a technique to detect change in LV volume and EF, both in the context of clinical research and for patients in a clinical setting. High reproducibility (low SD) leads to a greater reproducibility of observed changes in the LV volume, and therefore a smaller sample size for CMR studies can be used. The impact of smaller sample size means that studies can be completed faster and potentially at reduced cost.

It should be noted that this work was performed on healthy volunteers. Data acquired on patients may be of poorer quality alignment. In future work we intend to repeat the experimentation with patient data to confirm that similar results can be achieved.

In this study, a rigid transformation estimate was used to correct for differences in heart position due to respiration. In reality, the movement of the heart due to different breath-hold positions is not only limited to rigid motion, There will also be extended degrees of transformation (affine and non rigid parameters) [3]. The rigid transformation estimate was chosen for this work as the authors believed it could achieve good alignment accuracy without the added computational expense of more sophisticated transformation estimates. In future work, it would be of interest to investigate the optimum degrees of freedom to achieve good alignment accuracy whilst minimizing the computational expense.

To reconstruct the SA images after registration, each of the misaligned stacks were transformed using the registration transform into a regularly spaced voxel grid using linear interpolation. In such a reconstruction the data is potentially non-uniformly sampled onto a regularly spaced sampling grid; linear interpolation is far from the perfect interpolant. This could potentially lead to errors in LV volume analysis of the realigned data. It is recommended that in future work, more appropriate interpolation schemes should be investigated and used for a such a reconstruction.

## Authors' contributions

All authors have seen and approved the final version of the manuscript. AGC carried out the experimental studies, analysed the results, and drafted the manuscript. RJP carried out the segmentation and statistical analysis for the interstudy reproducibility study and aided AGC in finalizing the manuscript. JS participated in optimizing the registration algorithm. TN and DJH participated in the design and coordination of the study. RR and DLH conceived of the study, and participated in its design.
